# The Impact of Eltrombopag Administration on the Clinical Course of Severe Refractory Fatal Acquired Aplastic Anemia

**DOI:** 10.4274/Tjh-2013.0005

**Published:** 2013-09-05

**Authors:** Ayşe Işık, Eylem Eliaçık, İbrahim C. Haznedaroğlu, Salih Aksu, Nilgün Sayınalp, Yahya Büyükaşık, Hakan Göker, Osman Özcebe

**Affiliations:** 1 Hacettepe University Faculty of Medicine, Division of Hematology, Ankara, Turkey

Severe aplastic anemia (SAA) has an aggressive clinical course and represents a “difficult-to-treat” situation with current medications [1]. Eltrombopag is a c-mpl receptor agonist oral thrombopoietin-mimetic drug, mainly active in immune thrombocytopenic purpura (ITP) [2]. Single-agent oral eltrombopag produced hematological responses in 11 of 25 cases of aplastic pancytopenia, with trilineage responses observed in some, suggesting a stimulatory effect of early myeloid progenitors in a pilot clinical trial [3]. We would like to share our experience with eltrombopag in 2 patients with SAA refractory to conventional immunosuppressive treatment. Since the thrombopoietin/c-mpl receptor system is present in early hematopoiesis and in hematopoietic stem cells (HSCs) [4], SAA is an area of potential clinical application for thrombopoietin receptor agonists, including eltrombopag [5].

A 19-year-old male patient was admitted to our emergency room with the complaints of nasal bleeding and ecchymosis. His medical history was unremarkable. On physical examination, the patient was appropriately alert and oriented, and no abnormality was detected except for mucosal petechial hemorrhages and ecchymosis. Laboratory studies revealed pancytopenia with Hb of 6.4 g/dL, leukocyte count of 1.5x10^9^/L, platelet count of 4x10^9^/L, and reticulocyte count of 0.2%. His absolute neutrophil count was 0.5x10^9^/L. His coagulation profile was normal. The peripheral blood smear was consistent with the complete blood count, without any morphological abnormalities. Additionally, immunologic and virology tests were carried out, revealing no abnormality. He had a negative DEB test. We did not perform molecular genetic analysis tests for DKC1, TERC, or TINF2 because they are not available at our center.

After his diagnosis was confirmed with bone marrow aspiration and biopsy as aplastic anemia with bone marrow cellularity of <10% and without any evidence of dysplasia, 1 mg/kg steroid and 5 mg/kg cyclosporin were started.

During his follow-up, acute vision loss developed and retinal hemorrhage was detected; when his platelet count was below 20x10^9^/L despite platelet transfusions. Daily platelet transfusion was started in an attempt to raise the platelet count since sufficient response was not obtained with immunosuppressive treatment. With the diagnosis we started to test for human leukocyte antigen (HLA) typing, but because the test results take time, we first started cyclosporin and steroid then when we added ATG to patients medication.

Following horse ATG at a dose of 40 mg/kg for 4 days, the patient became more cytopenic and needed more frequent transfusions, especially platelet suspensions. Despite multiple platelet transfusions, his platelet counts remained below 50x10^9^/L and so eltrombopag at 50 mg/day was begun (Figure 1). Although eltrombopag is not licensed for use in SAA cases, we provided the drug via application to the Ministry of Health for off-label use of eltrombopag. Both patients signed an informed consent form. During the follow-up period, profound swelling developed in the left part of his face. Computed tomography (CT) scanning was consistent with mucormycosis. Antifungal treatment was begun and he underwent surgical debridement. One week after the eltrombopag initiation, the patient’s platelet counts remained below 50x10^9^/L without any transfusion support, but we did not notice marked changes in Hb or neutrophil counts. However, septic shock complicated the clinical picture and the patient died. 

A 44-year-old female patient with a history of bronchial asthma was admitted to our emergency service with the complaints of epistaxis, bleeding from the ears, and vision loss. Her complete blood count revealed Hb of 6.2 g/dL, leukocyte count of 2.2x109/L, platelet count of 4x10^9^/L, and reticulocyte count of 1% with normal coagulation parameters. Her neutrophil count was 0.7x10^9^/L. Her MRI showed subdural hematoma, which was more prominent in the right parietal and left frontoparietal regions, also accompanied by subarachnoid hemorrhage. She was counseled that neurosurgery and surgical procedures were not needed, and dexamethasone (16 mg/day) as an antiedema therapy and phenytoin were recommended. Her bone marrow aspiration and biopsy were consistent with aplastic anemia, with bone marrow cellularity of <10% and no evidence of dysplasia. We did not perform a DEB test or molecular genetic analysis for DKC1, TERC, and TINF2, as those tests are not available at our center. We started cyclosporine A in addition to dexamethasone. Because of her intracranial hemorrhage, we did not plan ATG administration in order to avoid profound thrombocytopenia due to the drug itself after intracranial hemorrhage. During this period, she remained profoundly thrombocytopenic, requiring twice daily platelet transfusions to achieve a platelet count over 100x10^9^/L, and we started eltrombopag at 50 mg/day (Figure 1). Although we obtained a relative response to platelet transfusion we could not achieve platelet count of 100x10^9^/L, which is recommended for her intracranial hemorrhage, we started eltrombopag. Following the initiation of the drug, her liver function test results elevated and skin rashes appeared. Skin-punch biopsy was consistent with drug eruptions. Following the addition of oral and topical antihistaminic drugs, her skin rashes disappeared. Her liver function tests returned to normal with a dose reduction to 25 mg/day. After then increasing the dose again to 50 mg/day, her liver function values remained controlled. Two weeks after the initiation of the eltrombopag, her platelet transfusion requirement was reduced with platelet counts reaching the 80.000/µL range without transfusions. As enough time was not passed for occurence of response to immunsuppresive therapy, we attributed the relative recovery in platelet counts and decrease in platelet transfusion requirement to eltrombopag. After eltrombopag administration we did not notice marked changes in Hb or neutrophil counts. Her follow-up MRI showed regression of the intracranial hemorrhage. Her donor screening tests revealed a related HLA-matched donor, and accordingly allogeneic HSC transplantation was planned. During her hospitalization, abdominal pain and fever developed. Her abdominal CT scan was consistent with typhlitis without perforation. Antimicrobial therapy was started. After 2 days, she became septic and died. 

In summary, both cases of SAA presented here suggest that eltrombopag could reduce transfusion requirements in patients with platelet transfusion-dependent aplastic anemia. However, the drug had no impact on the morbidity or mortality in our patients with SAA. Informed consent was obtained.

Olnes et al. obtained clinical response in aplastic thrombocytopenia via the administration of eltrombopag, the c-mpl agonist oral thrombopoietin mimetic [3]. Likewise, Komatsu et al. previously disclosed that platelet counts were increased after intravenous PEG-reHuMGDF, a truncated recombinant version of c-mpl ligand (thrombopoietin), in aplastic anemia and myelodysplasia. The peak platelet level has been observed within 5 to 6 weeks after the initiation of PEG-reHuMGDF treatment [5]. The efficacy of thrombopoietins for the reversal of thrombocytopenia requires the presence of hematopoietic and megakaryopoietic progenitors in the bone marrow (BM). Thus, thrombopoietins are active in diseases with BM megakaryocyte mass such as ITP and thrombocytopenia following nonmyeloablative chemotherapy. However, thrombopoietins are ineffective in thrombocytopenias due to myeloablation as a consequence of the inherent kinetics of thrombopoiesis [5]. Therefore, quantification of BM megakaryocyte mass before eltrombopag therapy can predict the response to thrombopoietins in thrombocytopenia. The number of BM megakaryocytes was expressed as the number of GPIIb/IIIa-positive cells per cellular area, which was calculated by subtracting the fatty area from the total area in a previous study [6]. Similar techniques could be applied to the paraffin-embedded BM sections of patients with aplastic pancytopenia to search BM megakaryocyte masses for the prediction of eltrombopag-respondent versus nonrespondent cases [3].

## CONFLICT OF INTEREST STATEMENT

The authors of this paper have no conflicts of interest, including specific financial interests, relationships, and/ or affiliations relevant to the subject matter or materials included. 

## Figures and Tables

**Figure 1:**
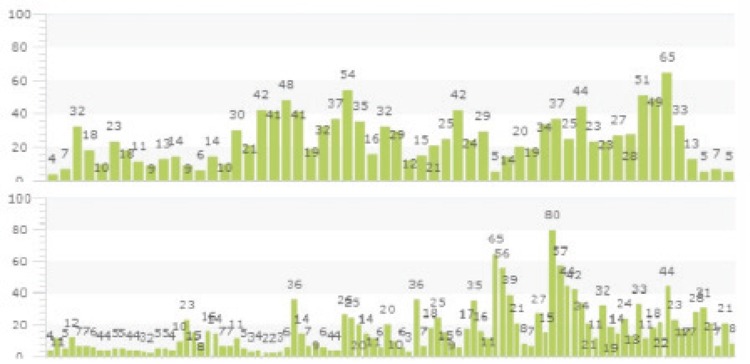
Platelet counts (x109/L) of Patient 1 (top) and Patient 2 (bottom). Eltrombopag was started on the days marked with arrows
